# Investigation of Fluid-Mechanical Interfaces (FMIs) for the Transmission of Oscillations in Hydraulic Fluid

**DOI:** 10.3390/s26196302

**Published:** 2026-10-06

**Authors:** Lorenzo Giunta, Jingqi Liu, Min Pan, Vimal Dhokia, Chris Bowen, James Roscow

**Affiliations:** Department of Mechanical Engineering, University of Bath, Claverton Down, Bath BA2 7AY, UKmp351@bath.ac.uk (M.P.); vd209@bath.ac.uk (V.D.); msscrb@bath.ac.uk (C.B.); jir24@bath.ac.uk (J.R.)

**Keywords:** energy harvesting, fluid-mechanical interface, piezoelectric

## Abstract

This study investigates fluid-mechanical interfaces (FMIs) for transmitting pressure oscillations in a hydraulic fluid to a piezoelectric stack, with the aim of supporting self-powered sensing and energy harvesting in hydraulic systems. A hydraulic test rig was employed in which a pump, switching valve, pressure sensors, and a piezoelectric stack were coupled through stainless-steel diaphragms of varying thickness (0.10 to 0.80 mm, 48 mm diameter). The effect of maximum pump pressure (20–50 bar) and valve switching frequency (1–50 Hz) on force transmission was evaluated, and diaphragm integrity was assessed after testing. The results demonstrate that diaphragm thickness strongly influences transmissivity under cyclic hydraulic loading. For the stainless-steel diaphragms tested, a low thickness of 0.25 mm provided a favourable balance between safety and force transmission, performing essentially as well as the thinnest 0.10 mm diaphragm while offering improved mechanical robustness. Thicker diaphragms up to 0.80 mm remained viable for sensing applications where maximal transmissivity is less critical. The findings suggest that FMI geometry can be tuned to improve energy transfer to embedded transducers while maintaining hydraulic integrity. Further work is needed to assess long-term durability under extended cyclic loading and to adapt the design for higher-pressure applications.

## 1. Introduction

The digitalisation of hydraulic valves, facilitated by integrated, self-powered sensors and real-time feedback and control, has the potential to significantly reduce the energy consumption of these widely used systems. Recent research has increasingly focused on harnessing mechanical energy from pressure fluctuations, vibrations, and hydraulic ripples for the purpose of powering sensor networks and embedded devices autonomously, a need that has gained urgency alongside the rise of the Internet of Things (IoT) and remote condition monitoring [1,2]. To enable optimal performance, there is a need for the fluid-mechanical interface (FMI) between any hydraulic system and sensor technology to be carefully designed to ensure longevity of both the hydraulic component and the sensor, whilst also enabling stable sensor performance. This paper aims to provide new insights into how FMIs can be designed to maximise the transmission of energy from hydraulic fluid into a sensor or energy harvester, thereby improving the performance and reliability.

In many hydraulic systems, traditional battery-powered or wired sensors present maintenance and environmental challenges, including the need for frequent battery replacements in hard-to-access locations [3,4]. Energy harvesting solutions that convert ambient mechanical energy into electrical energy could circumvent these issues by enabling energy autonomy in devices, reducing the environmental impact, and supporting continuous operation of IoT nodes [5,6]. Notably, dielectric materials and microelectromechanical systems (MEMS) technology have become foundational for sensor development, facilitating the dynamic monitoring capabilities of MEMS-based sensors. Of particular note is that those leveraging piezoelectric, triboelectric, and capacitive transducers have shown potential for capturing pressure and vibration energy, which can then be converted to electrical power for IoT applications and wearable devices [7,8]. Recent advancements also emphasise the potential of multi-source harvesting, which combine ambient energy sources such as radio frequency (RF), thermal, and mechanical energy into integrated solutions for robust, battery-free systems [9]. To date, a limitation to the widespread adoption of these technologies is linked to the minute amounts of power generated by these systems, which is often insufficient to power sensors continuously [3,10]. By improving the transmissivity of FMIs, it may be possible to improve the efficiency of the energy harvesting process while, simultaneously, guaranteeing the integrity of the hydraulic system to prevent leakage or risk failure. The benefits of improved transmissivity are not limited to improved energy harvesting; they will also benefit future researchers looking for novel ways to integrate pressure sensing technology into hydraulic systems such as by integrating sensors into manifolds, for example, through the use of additive manufacturing.

### 1.1. Background

Piezoelectric materials have become a key focus in the field of hydraulic energy harvesting due to their capacity to convert mechanical strain from pressure fluctuations into electrical energy, providing a sustainable power source for low-power electronic devices. However, hydraulic systems introduce unique challenges for piezoelectric sensors, such as material robustness, to prevent the leakage of hydraulic fluid, and maintaining high sensitivity in diverse pressure environments [11,12,13].

Skow et al. [12] designed a functional energy harvesting prototype that used a piezoelectric stack isolated from hydraulic fluids by means of a mechanical interface. By analysing piezoelectric stack properties and optimising inductive and resistive loads, they improved the power density of the hydraulic pressure energy harvester (HPEH), achieving milli-Watt power levels. These devices showcase the potential of piezoelectric integration in hydraulic systems, as they provide low-power generation from off-resonance pressure excitation. However, the design of the FMI was not a primary consideration in their paper, with the extraction of energy from the hydraulic fluid maximised by tuning and optimising the piezoelectric stack itself. The paper did note, however, that the ratio between the area of the FMI in contact with the fluid and the cross-sectional area of the piezoelectric stack plays a role in the amount of energy extracted. Furthermore, the authors highlighted that the fluid pressure oscillation frequency will likely be below the fundamental frequency of the piezoelectric stack (which is often in the kHz range, as opposed to the fluid pressure, which is generally an order of magnitude lower). This has a side effect of limiting electrical resonance within the system.

In recent work, Shi et al. [14] explored the energy harvesting capabilities of piezoelectric energy harvesters under varying hydraulic conditions, examining the effects of different electrical connection modes, including single output, series, and parallel modes, on energy conversion efficiency. Their study revealed that the pressure differential (amplitude) played a more critical role in energy output than the static pressure, i.e., the preload acting on the piezoelectric onto which the dynamic pressure is superimposed. Shi et al.’s [14] findings indicate that when exploring the options for FMIs, it is important to explore a wide pressure differential which, subsequently, means designing FMIs that are capable of resisting high pressures and repeated pressure cycling.

Sob and Pita [15] simulated energy harvesting in shock absorbers, showing that hydraulic flow and turbulence directly affect power output. Factors such as fluid viscosity, velocity, and damping pressure significantly influence the energy harvester’s capability to convert oscillatory motion within the hydraulic fluid into electrical energy. Their research, being purely based on simulation, did not elaborate on the actual design of the HPEH to transfer the kinetic energy from the hydraulic fluid into the energy harvester for their use case.

Whilst the general design and configuration of HPEHs have been discussed, to date there has been little focus on the FMI with respect to its potential key role on efficiently harnessing hydraulic pressure fluctuations and improving the performance of a piezoelectric sensor or energy harvester. Despite the lack of focus on the development of FMIs in the general energy harvesting literature, there are recent advances which demonstrate that innovative FMI structures and configurations can significantly improve the performance of piezoelectric and other energy harvesting technologies by amplifying and modulating pressure fluctuations.

#### 1.1.1. Resonators

For example, Lechuga Aranda et al. [16] developed a novel space-coiling resonator, designed to optimise acoustic gain, which enhances a pressure fluctuation energy harvester’s efficiency. This resonator design, intended to function at lower frequencies (<1000 Hz), outperformed traditional Helmholtz resonators in compactness and performance, indicating a promising direction for FMI designs that maximise energy harvesting in compact systems. However, this system was more complex than a simple edge-clamped compliant diaphragm and required considerably more space to house it. This makes it an unattractive solution for miniaturisation applications. Furthermore, the manufacture of such a complex FMI requires specialised machinery and adds considerably to the cost of implementation.

Cao et al. [17] investigated methods to enhance FMI efficiency by designing a force-amplified piezoelectric stack energy harvester that captured pressure fluctuations within hydraulic pipelines. A key innovation was a force amplifier that augmented energy transfer efficiency, which is particularly useful in low-frequency environments typical of hydraulic systems. Experimental validation of this approach demonstrated an ability to support self-powered sensor networks in pipeline health monitoring applications, which is increasingly relevant for sustainable infrastructure maintenance. However, their research did not focus on the FMI itself and focused merely on the force amplifier.

Li et al. [18] focused on optimising the energy transfer through integrated circuit interfacing to improve FMI efficiency. They reported advancements in piezoelectric energy harvesting, particularly for low-power applications. Techniques such as maximum power point tracking and self-powered circuits, their proposed integrated circuits improve overall system sensitivity and energy conversion efficiency. By promoting circuit integration and reducing parasitic energy consumption, these proposed interfacing solutions align with HPEH goals of creating autonomous, low-power devices that can operate effectively within fluid power systems.

#### 1.1.2. Diaphragms

In contrast, Xiao et al. [19] looked specifically at using a circular edge-clamped flat plate diaphragm as an FMI for piezoelectric harvesters. This configuration allows for detailed control of the force–deflection relationship in response to fluid pressure ripples, improving energy output by leveraging nonlinear dynamics. Their model, validated experimentally, demonstrated optimal energy conversion efficiency when tuned to specific vibrational amplitudes, highlighting how FMI structural configurations can influence energy harvesting performance through frequency response and load optimisation. However, the verification of the model presents some limitations for the applicability of the findings in hydraulic systems. Firstly, the validation was undertaken using an electric shaker rather than hydraulic pressure to load the piezoelectric stack. This is an important departure as the findings do not take into account the potential subtleties of adequately sealing the hydraulic fluid nor does the electric shaker approach allow for the secondary vibrations inherent to a hydraulic pump to be introduced. More importantly, the model and validation assumed that the force from the shaker is being applied to the piezoelectric stack directly. The edge-clamped plate acts as a backing to hold the piezoelectric stack in place. Essentially, in their study, the piezoelectric is clamped between the shaker and the diaphragm. This resulted in the efficiency of the harvested energy decreasing with decreasing diaphragm thickness as the thinner diaphragm deflected more, thereby reducing the force transmitted to the piezoelectric stack itself. However, in a hydraulic system, where the diaphragm would have the dual purpose of isolating the hydraulic fluid from the piezoelectric stack and transmitting pressure from the fluid into the piezoelectric stack, the forces will, by necessity, be acting through the diaphragm [20,21]. This implies that a thinner diaphragm would result in more efficient power transmission but additional verification; building on the foundations provided by Xiao et al. [19] is necessary to understand the optimal design of FMIs.

#### 1.1.3. Other Fluid-Mechanical Interfaces

Research into FMIs has increasingly focused on the testing and validation of energy harvesting devices to optimise design and functionality under real-world conditions, highlighting the need to not only focus on analytical approaches. Recent studies have experimented with a range of setups and models. For instance, Ko et al. [22] developed a pico-scale Francis turbine and validated its performance in controlling pressure regulation and power generation through not only a computational fluid dynamics model but also via a custom experimental setup. This FMI design enabled flexibility by adjusting guide vane angles, showing a consistent agreement between model predictions and experimental results. These successes notwithstanding, Giunta et al. [23] highlighted the absence of standardised testing methods for HPEHs and proposed a test platform that adapts to high pressures and various FMI configurations, thereby attempting to tackle some of the shortcomings identified.

#### 1.1.4. Summary

In summary, the integration of FMIs into hydraulic energy harvesting devices remains a dynamic area of research. Current approaches reveal that the interplay between fluid dynamics, FMI structure, and interfacing circuitry can greatly influence overall energy conversion efficiency. The studies analysed also highlight an existing knowledge gap around the optimal use of FMIs.

Resonators can enhance pressure fluctuation transmission and energy harvesting performance while offering compact alternatives to conventional designs, but their increased structural complexity, space requirements, and specialised manufacturing demands can limit their suitability for miniaturised, low-cost FMI applications. In contrast, diaphragm-based FMIs offer a simple configuration capable of efficient pressure-to-mechanical energy transmission and tuneable nonlinear behaviour, but their performance is strongly dependent on diaphragm thickness and loading conditions, with existing validation not fully representative of the coupled pressure, sealing, and vibration dynamics encountered in hydraulic systems. Other FMI configurations offer flexibility and the potential for effective energy transfer across a range of operating conditions, but their performance can be highly dependent on system configuration and operating conditions, with the lack of standardised testing methods making direct comparison between designs difficult.

These studies contribute to a growing body of knowledge that can inform future designs aimed at improving FMI performance across a range of applications, from automotive damping systems to sustainable infrastructure monitoring.

### 1.2. Contribution to Knowledge

This study aims to build upon existing research by investigating FMIs specifically designed to maximise the transmission of pressure fluctuations and oscillations within hydraulic fluid. By evaluating FMI designs for optimised energy transmission, this research aims to develop a scalable and replicable approach to the design of FMIs that will aid future researchers in the development of the next generation of low-maintenance, self-sufficient sensor and energy harvesting systems. The specific contribution to knowledge this paper provides is an exploratory overview of the current landscape of FMI designs. The knowledge contained in this paper will assist future researchers as well as industry professionals in understanding the impact of FMI thickness on energy transmission efficiency. Furthermore, the paper provides a cautionary overview of the dangers that arise from FMIs that are too thin.

This paper continues by discussing an experimental approach to design and test FMIs with a range of thickness levels when subject to pressure ripple conditions. Results are presented to demonstrate how pressure fluctuations at various frequencies impact the energy transmitted to the piezoelectric stack used as a sensor. In addition, mechanical damage to the FMIs during testing is evaluated to highlight the potential risk of failure caused by the cyclic loading.

## 2. Materials and Methods

### 2.1. Experimental Setup

**Figure 1:**
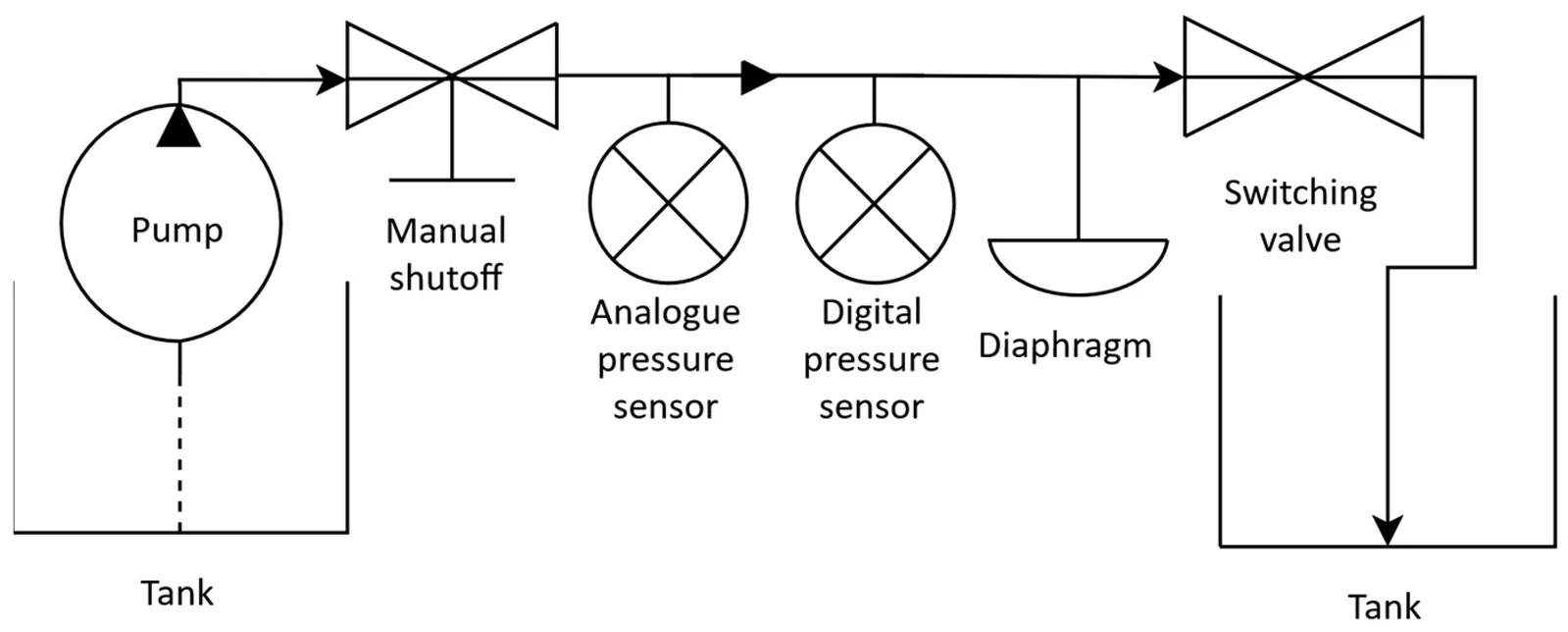
Flow diagram for hydraulic test rig.

Figure 1 shows the flow diagram of the system used for applying pressure to the piezoelectric stack, where a pump was used to circulate hydraulic fluid through the circuit. The fluid flows through a manual shutoff valve that allows for the isolation of the pressure vessel where the diaphragm and piezoelectric stack are housed. The fluid then passes through two pressure sensors; a digital sensor (Mesurex, Saint-Arnoult en Yvelines, France, Part Nr: PSM5C350B, accurate to ±0.25%) is used for data capture, and an analogue sensor is used for instantaneous overview of the system condition. The fluid then passes through the switching valve (Parker DF Plus D1FPE50MA9NB00), which is electrically operated by means of a signal generator to permit or restrict the flow of hydraulic fluid, allowing for the buildup of pressure and the deformation of the diaphragm. Lastly, the fluid passes back to the tank.

**Figure 2:**
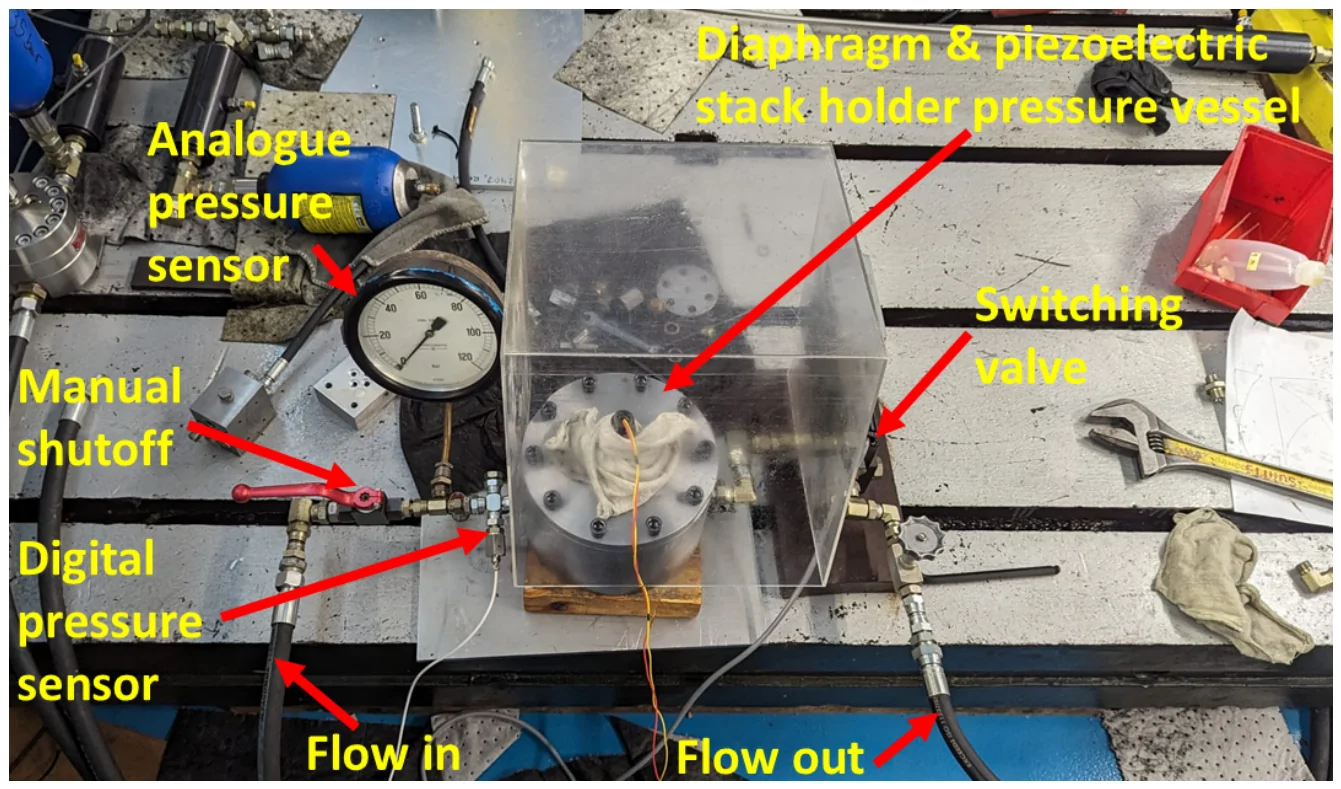
Annotated hydraulic rig setup.

**Figure 3:**
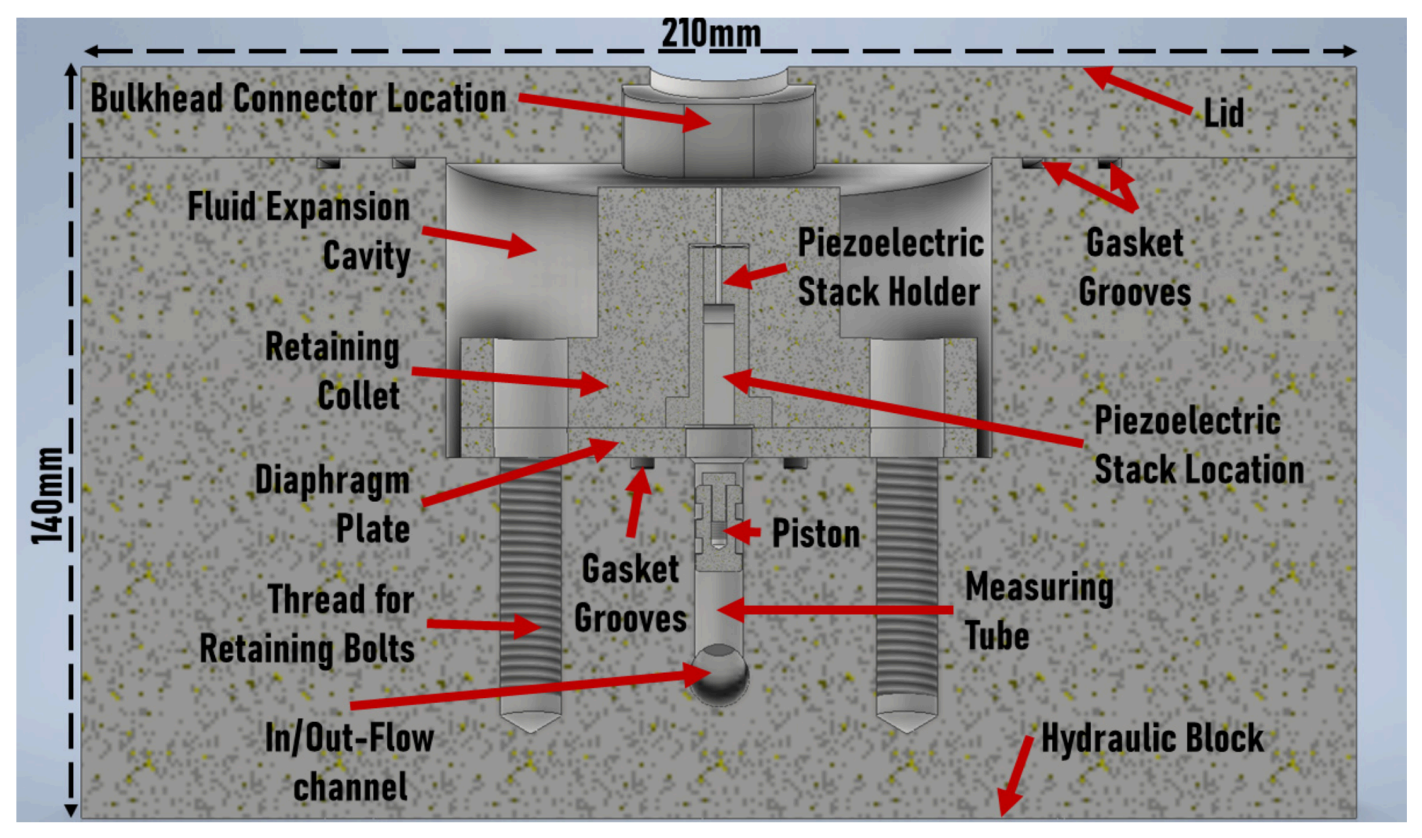
Pressure vessel internal design using the geometry and setup detailed by Giunta et al. [23].

Figure 2 shows how the flow diagram discussed in Figure 1 was implemented. As can be seen from the figure, a protective cover was kept on top of the pressure vessel in order to minimise the risk of injury in case of a leak. The pressure vessel that was used to hold the piezoelectric stack and diaphragms was based on the pressure vessel design proposed by Giunta et al. [23]. The design, shown in Figure 3, allows for multiple sizes of piezoelectric stacks to be tested as well as diaphragms of different thicknesses. In this work, diaphragms of different thicknesses with a single piezoelectric stack were tested.

As can be seen from Figure 3, the pressure vessel is composed of three main components, plus the diaphragm itself; these are (i) the retaining collet, (ii) the hydraulic block, and (iii) the lid. The hydraulic block acts as a conduit for the hydraulic fluid with a straight channel that allows unimpeded flow. A measuring tube branches off from this channel and goes to the diaphragm. The diaphragm is sandwiched between the hydraulic block and the retaining collet and is sealed by means of a rubber gasket to prevent hydraulic fluid from leaking. The retaining collet holds the piezoelectric stack holder and is secured to the hydraulic block by means of six bolts, which can be torqued to different levels to increase the preload on the piezoelectric stack. Finally, the lid seals the whole pressure vessel, allowing only electrical signals from the piezoelectric stack to be transmitted by means of a bulkhead connector. The diaphragm is installed within the pressure vessel. Thereafter, the piezoelectric stack is placed within the retaining collet and the collet is pressed down onto the diaphragm and bolted in place using a torque wrench to ensure even pressure application. Once the system is fully sealed, the switching valve is powered on to generate a pressure ripple on the diaphragm. The flowrate control was used to ensure a steady flow of fluid into the system and to guarantee that the pressure ripples generated were as close to a sinusoidal wave as possible.

### 2.2. Experimental Parameters

The experimental parameters explored in this study explored the effect of maximum pump pressure (bar), frequency (Hz) of the valve opening and closing, and diaphragm thickness (mm). The maximum pump pressure was varied from 20 to 50 bar in 10 bar increments and tested at frequencies of 1, 5, 10, 20, and 50 Hz. Stainless-steel diaphragms with thicknesses of 0.10, 0.15, 0.20, 0.25, 0.30, 0.40, 0.50, 0.65, and 0.80 mm were evaluated. These have resonant frequencies ranging from 117 Hz (0.1 mm thick diaphragm) to 936 Hz (0.8 mm thick diaphragm). All diaphragms were made of stainless-steel shim taken from the same batch and had a diameter of 48 mm. The diaphragms were manufactured using a punch to guarantee the size of each diaphragm. These diaphragms were clamped in place with 25 Nm torque that was applied using a calibrated torque wrench.

The following variables were controlled across all experimental conditions. The piezoelectric stack employed as the sensor or harvester (Noliac, Lisle, IL, USA, NAC2013-H20 which has a minimum resonance of 22 kHz, vastly higher than the highest test frequency used) was kept constant and the same stack was used in all experiments. This stack was connected to an oscilloscope (Keysight, Santa Rosa, CA, USA, DSOX1204A) in open circuit configuration with an internal impedance of 1 MΩ. The oscilloscope collected data at a sampling rate of 25 kHz over a 2-s period once a steady state was achieved. A driving voltage from a signal generator (3v PtP) with a sinusoidal waveform was used to actuate a hydraulic valve to create the hydraulic pressure ripples.

In order to simplify the data collection and to ensure that all data was synchronised to the same time start point, an oscilloscope was used to capture the pressure and voltage readings from the pressure sensor and piezoelectric stack. The data captured by means of the oscilloscope included a timestamp, voltage from the pressure sensor, voltage from the piezoelectric stack, and the signal generator output used to drive the switching valve. Pressure was calculated from the voltage measured from the pressure sensor in bar using the manufacturer’s calibration.

### 2.3. Data Processing

The piezoelectric voltage coefficient, which is the voltage generated per unit stress applied to a piezoelectric sensor, is given by (1):

(1) $$g_{33}=\frac{d_{33}}{\varepsilon_{33}^{\sigma }}$$

where *d*_33_ is the piezoelectric charge coefficient (units: C/N) and $\varepsilon_{33}^{\sigma }$ is the permittivity of the material under constant stress (units: F/M). The open circuit voltage generated by a piezoelectric stack under an applied stress amplitude (namely the change in pressure applied to the piezoelectric stack) ∆*σ_j_* (N/m^2^) is given by (2):

*V_oc_* = *g*_33_ ∙ ∆*σ_j_* ∙ *t* (2)

This ignores only loss of charge and reduction in voltage, due to the impedance of any measurement equipment such as the oscilloscope. This is a valid assumption here as the time constant *τ* = *RC* of the piezoelectric (*C* = 1630 nF) and oscilloscope (R = 1 MΩ), which determines the time for charge to dissipate, was 1.63 s, i.e., significantly larger than the data sampling rate. Equation (3) expands on the above formulae by describing the open circuit voltage obtained from a piezoelectric stack. It does so by substituting the thickness *t* of a single piezoelectric element with the total length of the piezoelectric stack (*L*, metre) and dividing this by the total number of piezoelectric elements (*n*, unitless), which would logically result in the thickness of an individual piezoelectric element; hence, *L* = *n.t*. In addition, Equation (3) substitutes the change in stress applied to the piezoelectric element (∆*σ_j_* Newton/metre^2^) with the change in force applied to the piezoelectric element (*F_ripple_*, Newton) divided by the cross-sectional area of the piezoelectric stack (perpendicular to the application of the force) (*A_s_*, metre^2^). This allows for the equation to be written in terms of change in force applied to the piezoelectric stack.

(3) $$V_{\mathrm{oc}}=\frac{d_{33}}{\varepsilon_{0}\cdot \varepsilon_{33}^{\sigma }}\cdot \frac{F_{\mathrm{ripple}}}{A_{s}}\cdot \frac{L}{n}$$

Equations (4) and (5) describe how the peak-to-peak (PtP) voltage is calculated using the formula for *V_rms_* (Volt) (Equation (5)). The PtP voltage (*V_PtP_*, Volt) corresponds to the open circuit voltage generated by the piezoelectric stack as it is loaded. Thus, using Equation (3), it is possible to use the PtP voltage to understand the change in force applied to the piezoelectric stack between the minimal load condition and the maximum load condition.

(4) $$V_{\mathrm{PtP}}=2\cdot \sqrt{2}\cdot V_{\mathrm{rms}}$$

(5) $$V_{\mathrm{rms}}=\sqrt{\frac{1}{n}\sum_{i=1}^{n}x_{i}^{2}}$$

The open circuit voltage was used to calculate the load applied on the piezoelectric stack, motivated by the desire to reduce the effects of damping, frequency shift and electrical impedance matching that could otherwise result were the piezoelectric attached to a closed circuit [24]. The measuring equipment used to read the voltage output from the piezoelectric stack will not have had an infinite impedance; however, for the purposes of this experiment the impedance was assumed to be infinite.

## 3. Results

**Figure 4:**
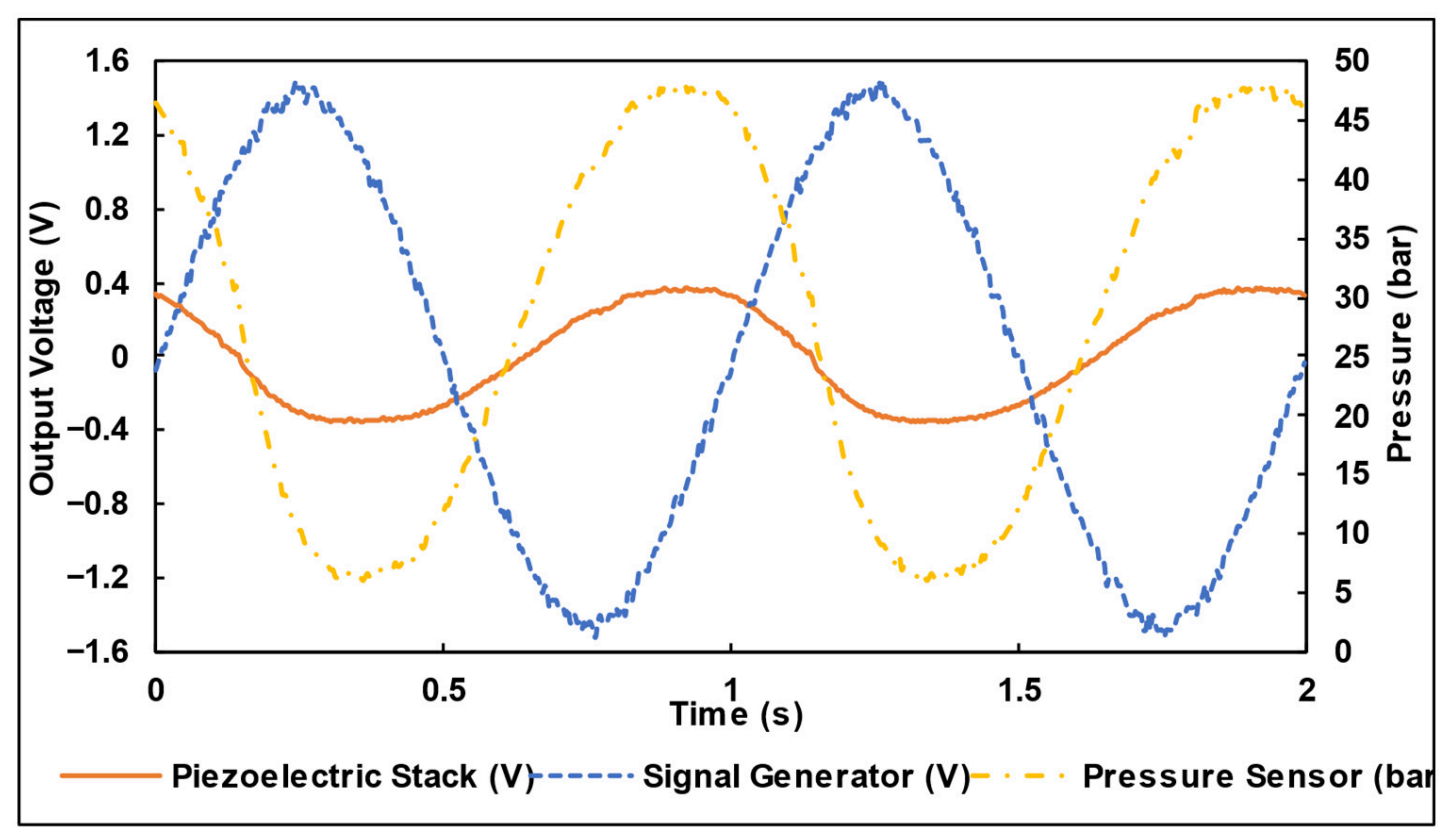
Data from experiment from piezoelectric stack, pressure sensor and signal generator to drive hydraulic valve with pressure at 50 bar, valve frequency at 1 Hz and a 0.80 mm diaphragm.

This section presents the results obtained from both the pressure sensor and the piezoelectric stack. Through an analysis of the output for the pressure and the subsequent voltage generated from the piezoelectric stack, it is possible to determine the transmissivity of the FMI as a function of thickness. Figure 4 shows example data for one condition. A sinusoidal wave is generated by the signal generator to control the opening and closing of the switching valve. This leads to a variation in the pressure, which is measured by both inline pressure sensor (yellow) and the open circuit voltage of the piezoelectric stack (orange). A good correlation between the response of the pressure sensor and the piezoelectric stack output was observed, and there is a delay between the maximum voltage from the signal generator being output and the peak pressure being reached.

**Figure 5:**
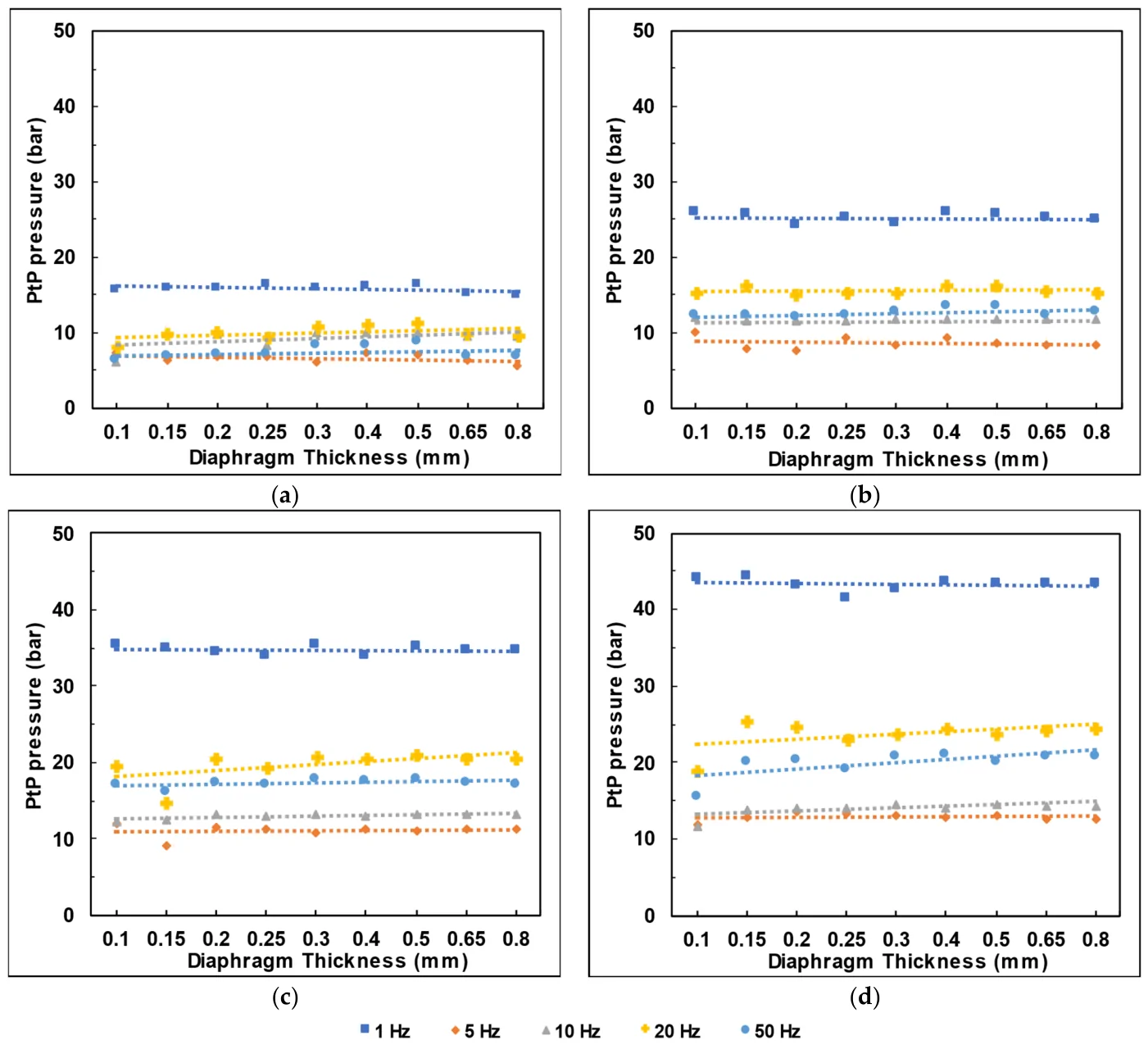
Peak-to-peak (PtP) pressure measured from the sensor for all frequencies and diaphragm thicknesses. Each graph was for a different maximum pump pressure. (**a**) 20 bar, (**b**) 30 bar, (**c**) 40 bar, (**d**) 50 bar.

The impact of diaphragm thickness on the transmission of mechanical energy from the hydraulic fluid into the piezoelectric stack was investigated. To perform this analysis, it was first necessary to ascertain whether the PtP pressure remained constant between diaphragm conditions. Figure 5 shows the PtP pressure for all the experiments, where each quadrant shows the PtP pressure measured with a different maximum pump pressure. From Figure 5, it is clear that PtP pressure was consistently highest in the 1 Hz condition. This outcome is expected, as the low switching speed allows sufficient time for the pressure to build up to the pump’s preset maximum and also permits a full drop to the minimum. In addition, PtP pressure increased as the maximum pump pressure was raised, which aligns with anticipated system behaviour. However, while the relationship between maximum pump pressure and PtP pressure was linear in all conditions, the rate at which the PtP pressure increased in relation to the maximum pump pressure was predicated on the switching frequency used. This is due to the aforementioned effect of faster switching frequencies not providing sufficient time for pressure to build up and come down; PtP pressure was not influenced by changes in diaphragm thickness, as expected.

**Figure 6:**
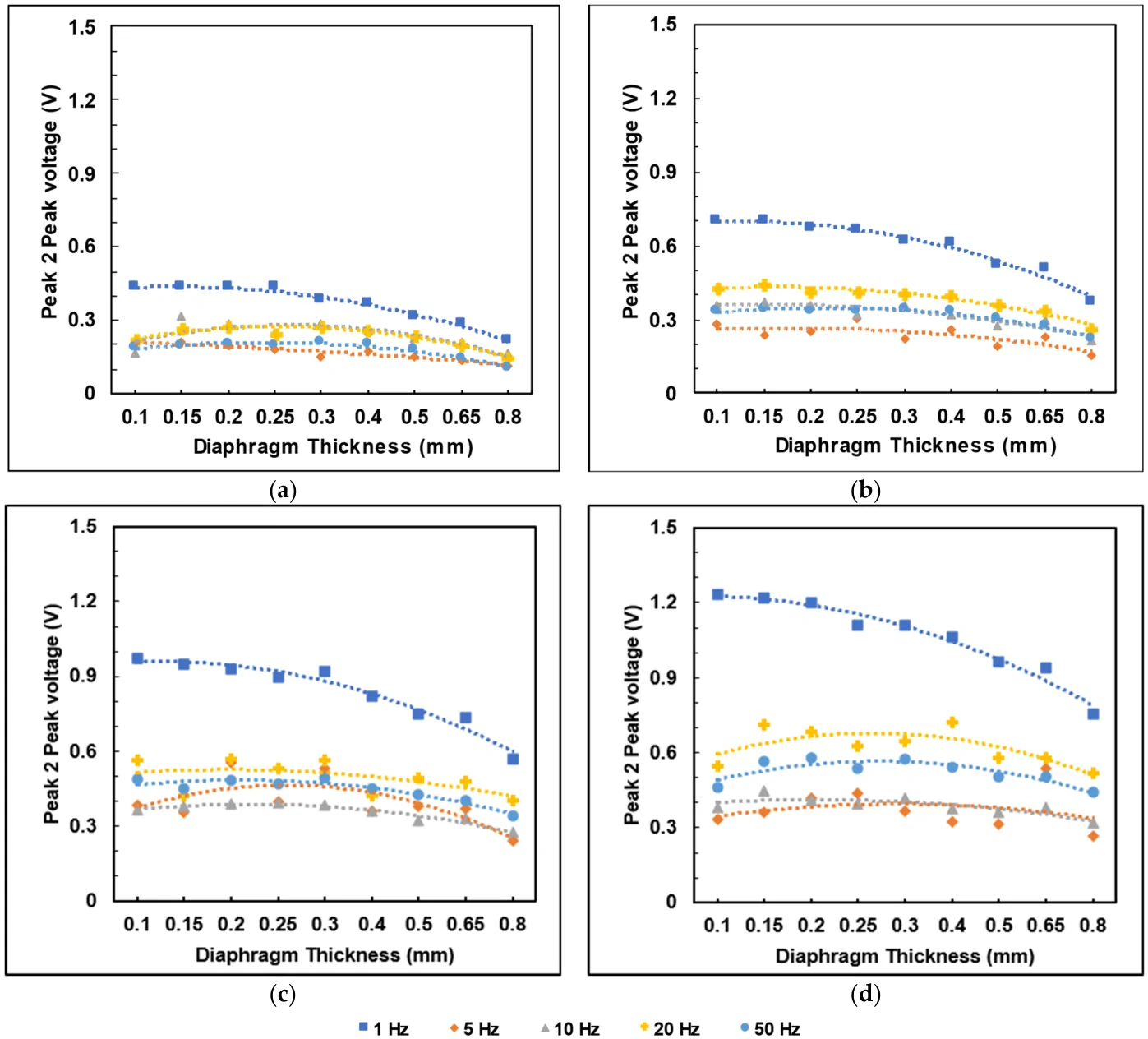
Peak-to-peak (PtP) open circuit voltage (V_OC_) measured from the piezoelectric stack for all frequencies and diaphragm thicknesses. Each graph represents different maximum pump pressures. (**a**) 20 bar, (**b**) 30 bar, (**c**) 40 bar, (**d**) 50 bar.

Figure 6 shows the measured open circuit voltage, V_OC_, measured from the piezoelectric stack. The voltage is generated due to the pressure differential caused by the oscillation of the hydraulic fluid and transmitted through the FMI to the piezoelectric stack. The results shown in Figure 6 align with the expected findings based on the PtP pressures shown in Figure 5. As expected, an increase in PtP pressure resulted in a corresponding increase in PtP voltage, as in Equations (2) and (3). At no point during the experiment was the piezoelectric stack depolarised, indicating potential for higher pressures for greater energy generation.

A variation in the FMI thickness did result in some attenuation in the open circuit voltage generated. However, as can be seen from a comparison of Figure 5 and Figure 6, this effect is most prominent when the PtP pressure is higher. This implies that at higher frequencies, the voltage loss may not be substantial enough to undermine the energy harvesting capability of the system even when very thick diaphragms (>0.65 mm) are used in high-pressure applications.

In addition, the voltage generation attenuation does not scale linearly with diaphragm thickness. Figure 6a shows no major difference in PtP voltage observed between the 0.1 mm and 0.25 mm diaphragms for the 1, 5 and 50 Hz condition and even shows a marginal improvement for the 10 and 20 Hz conditions. However, examination of Figure 6b–d reveals that the effect of diaphragm thickness on voltage attenuation appeared to be less pronounced at lower PtP pressures. Increasing maximum PtP pressure results in minor increases in attenuation across all conditions. This effect, however, remains relatively minor below the 0.25 mm thickness FMI. Nonetheless, even with the thickest diaphragms tested, energy was successfully transmitted from the fluid medium into the piezoelectric stack.

**Figure 7:**
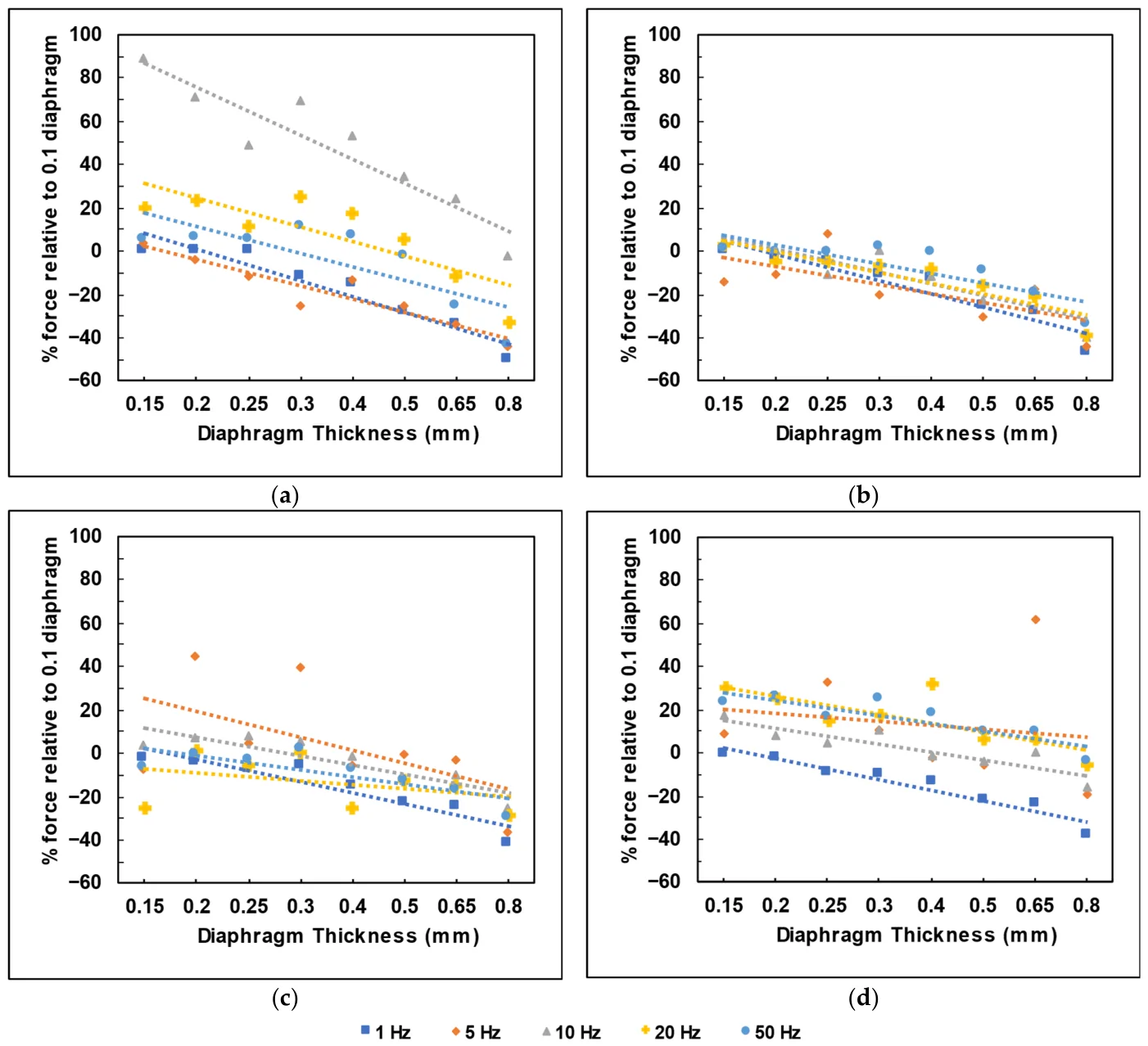
Force transmitted by a given diaphragm across a range of pressures and frequencies relative to the force transmitted by the 0.1 mm diaphragm. Each graph was for a different maximum pump pressure. (**a**) 20 bar, (**b**) 30 bar, (**c**) 40 bar, (**d**) 50 bar.

Figure 7 compares the force transmitted through the diaphragm, calculated using Equation (3) for each of the four pressure conditions. The force transmitted by a given diaphragm across a range of pressures and frequencies is plotted relative to the force transmitted by the 0.1 mm diaphragm for the same pressure and frequency condition and then normalised to zero, such that an equal force acting through a different diaphragm and test condition would equal zero. The increase in diaphragm thickness leads to a reduction in the force transmitted for all test conditions as is expected. However, Figure 7a shows the most significant decrease in performance measured. In addition, it is unclear why the transmission efficiency of the 10 Hz frequency is notably higher than in any of the other conditions, across all pressure ranges and diaphragm thicknesses. It is possible that this result is caused by resonance or that a data collection error at lower frequencies is to blame.

However, upon analysing Figure 7c,d, a similar behaviour can be seen for the 5 Hz condition, where a few specific diaphragm thicknesses (0.2 mm and 0.3 mm in Figure 7c, 0.65 mm in Figure 7d) result in much higher transmission of force compared to the other diaphragm thicknesses. This may be indicative of resonant behaviour, which would strengthen the argument that this is also occurring across all diaphragm thicknesses in the 10 Hz condition shown in Figure 7a.

**Figure 8:**
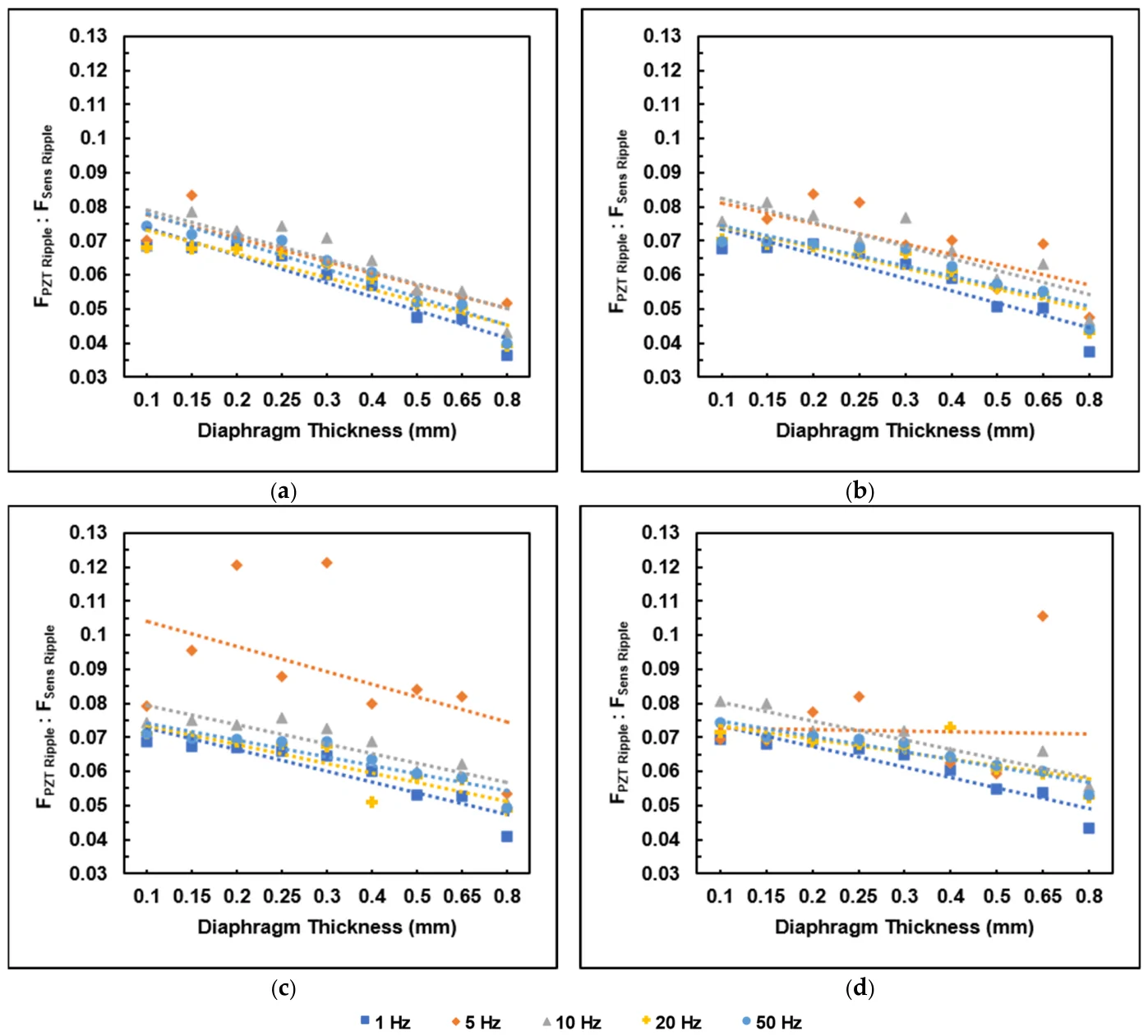
Ratio of force measured by sensor and force acting on the piezoelectric stack. Each graph was for a different maximum pump pressure. (**a**) 20 bar, (**b**) 30 bar, (**c**) 40 bar, (**d**) 50 bar.

Figure 8 shows the ratio of force on the piezoelectric stack and the force on the diaphragm (as measured by the pressure sensor). This ratio shows the transmissivity of force from the hydraulic fluid to the piezoelectric stack. As seen with Figure 7, it is clear that there is a direct relationship between the thickness of the FMI diaphragm and the force transmitted from the hydraulic fluid into the piezoelectric stack. The force transmitted between the 0.1 mm and 0.8 mm diaphragms is reduced by between 53% and 75%, with smaller reductions in force occurring where the maximum pump pressure was higher.

**Figure 9:**
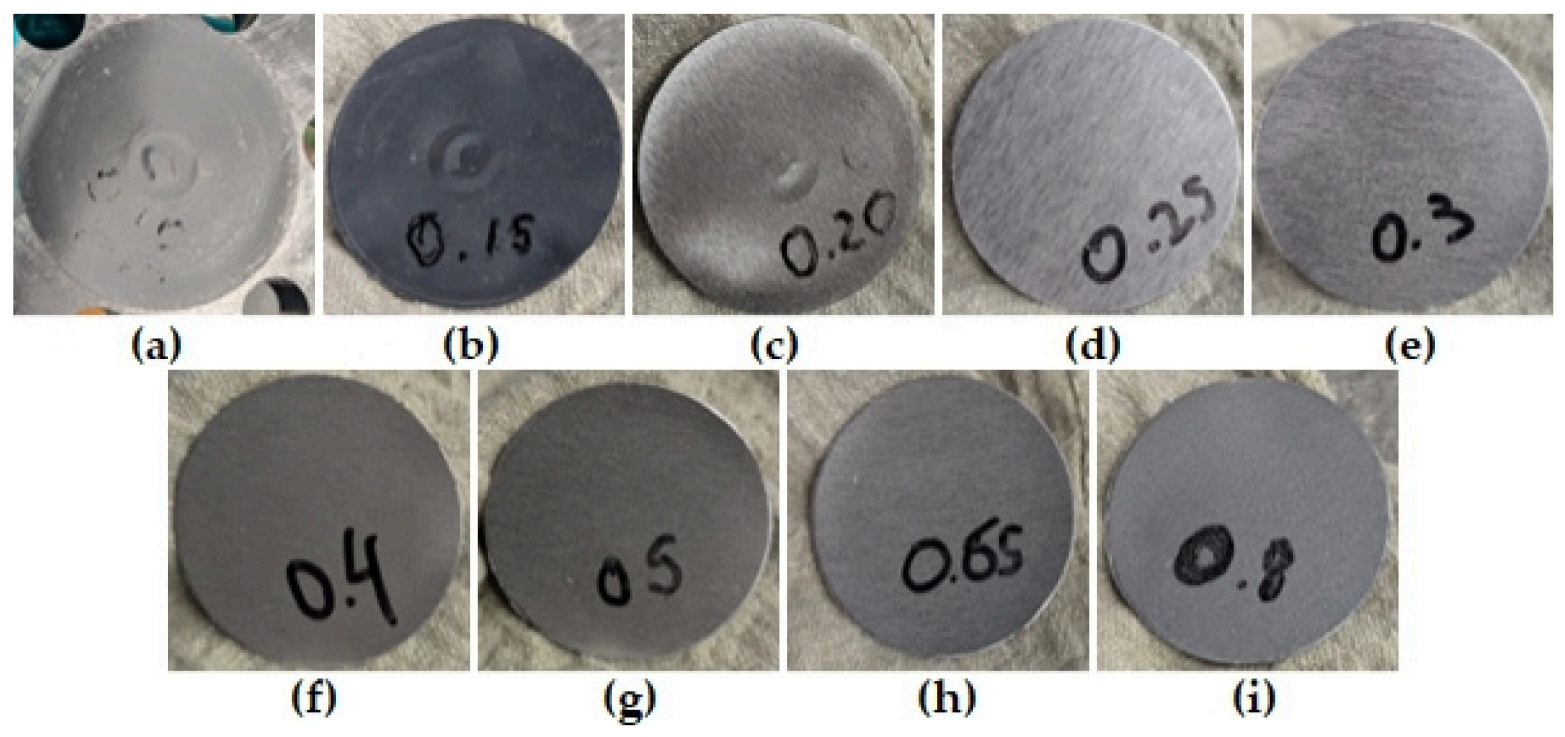
Diaphragms with diameter of 48 mm and a thickness of 0.10 mm to 0.80 mm (figure (**a**–**i**)) showing deformation patterns in the figure (**a**–**c**) discs where the metal has been bent around the piezoelectric stack.

Finally, Figure 9 highlights the effect that the pressure has had on the different diaphragms used. Diaphragms with a thickness of ***t*** = 0.1 mm to 0.2 mm showed permanent plastic deformation during the testing with a clear imprint left on them by the piezoelectric stack and holder. This effect is slightly less marked on the 0.2 mm diaphragm and is completely absent from any thicker diaphragms. This indicates that at the pressure ranges tested during this experiment, which reached 50 bar with a maximum PtP of 45 bar, the thinner diaphragms could have failed with repeated cycling and potentially leaked. This is especially important to consider when thinking about the long-term viability of the FMIs as, while no leakages occurred during experimentation, loading and unloading the diaphragm over the period of hours or longer could have resulted in failure.

## 4. Discussion

Based on the findings reported in Section 4, a number of clear trends emerge. The most important trend is the inverse proportionality between diaphragm thickness and force transmission. While this finding appears, prima facie, obvious, the value of the research presented within this paper lies primarily in highlighting that not only is this relationship linear, but the reduction in force transmitted is also relatively minor.

Indeed, as highlighted in Figure 7 and Figure 8, the force transmitted by the 0.8 mm diaphragm was reduced by between 24% and 47% compared to the 0.1 mm diaphragm for the same operating conditions. This finding is particularly valuable for the design and development of an FMI for sensor applications. While an energy harvesting system would naturally want to maximise the amount of transmissivity from a diaphragm, while still avoiding failure, the development of an FMI for sensor applications merely requires the FMI to not attenuate the signal so much that a measurement cannot be taken.

In addition, the figure highlights an interesting phenomenon observed at 10 Hz, where the transmission of force is notably higher than that of the other conditions. Similar behaviour was seen can be seen for the 5 Hz condition, where a few specific diaphragm thicknesses (0.2 mm and 0.3 mm in Figure 7c, 0.65 mm in Figure 7d) result in much higher transmissions of force compared to the other diaphragm thicknesses. This behaviour could be linked to resonance, but further analysis is necessary as the obvious sources of resonance, namely the piezoelectric stack and the diaphragms themselves, have notably higher resonant frequencies. Indeed, the piezoelectric stack’s resonance is 22 kHz, and the 0.1 mm diaphragm has a resonance of 117 Hz. With the resonant frequencies being so far out of the operating frequencies of the experiment, and the behaviour being present for all the diaphragms, it is less apparent what could be causing the resonance, if the behaviour is even caused by resonance at all. Further investigation to isolate the behaviour is clearly necessary. However, this does not detract from the findings, which clearly highlight that the diaphragm thickness influences energy transmission.

From this perspective, the research presented within this paper has shown that diaphragms of up to 0.8 mm are capable of transmitting sufficient force for measurement using a piezoelectric stack as a sensor, even at pressures as low as 5 bar, though based on the findings from Figure 6 it is likely that 0.8 mm is beginning to approach the maximum thickness for the FMI as a sharp decrease in PtP voltage generated can be seen from the 0.5 mm diaphragm onwards.

Figure 6 provides additional context for the development of FMIs for energy harvesting as well. As can be seen from the figure, there appears to be no substantial drop in the PtP voltage generated from the piezoelectric stack with diaphragms of 0.1 mm to 0.25 mm thickness. This, combined with the fact that the attenuation of the FMI appears to be less relevant at low pressures, indicates that a functional energy harvesting FMI can be made of up to 0.25 mm steel without any major loss in transmissivity of energy even at low-pressure oscillations (5 bar PtP with a minimum pressure of 5 bar).

This is particularly important due to the findings highlighted in Figure 9. Figure 9 provides clear data that diaphragms of less than 0.2 mm are unlikely to be able to survive long-term operation at the peak pressures used in this study (50 bar). This raises serious safety concerns and highlights the dangers involved; while no leaks were detected and appropriate safety precautions were taken during this experiment, no accelerated life testing was undertaken. Combined with the clear indentations and deformations on the diaphragms shown in Figure 9, and it becomes apparent that the 0.1–0.2 mm diaphragms likely would have failed and leaked if operated for significant amounts of time. This implies that for energy harvesting purposes, a stainless-steel diaphragm should be designed with a thickness between 0.2 mm and 0.25 mm for the conditions examined here.

However, the open circuit voltage indicates that the maximum stored electrical energy in the piezoelectric, calculated from Equation (6), is around 1 micro-Joule for the highest measured voltages above 1 V (given that C = 1630 nF for the Noliac NAC2013-H20). Previously, Li et al. [25] estimated the extractable energy to be ~50% of the stored electrical energy using an impedance matched circuit. Taking a conservative estimate of 10% extracted energy would give an estimated harvested energy of 0.1 micro-Joule per cycle. This is a rather low value, and could highlight the need for either additional research, e.g., force multiplication, or the relegation of this technology purely as a sensor in V_OC_ mode.

(6) $$U=\frac{C{\cdot V}^{2}}{2}$$

However, based on the findings from Figure 6, which indicate that the potential gains in transmissivity due to a thinner diaphragm in this range are insignificant, a 0.25 mm diaphragm would be optimal for long-term use in an operational environment that resembles the cases evaluated in this study due to the safety concerns of using anything thinner, as this diaphragm thickness showed no deformations after testing, though accelerated life testing should be undertaken to guarantee this. Indeed, additional testing would have to be undertaken to confirm the safety of the diaphragm under cyclical loading and at potential spike pressures to guarantee that deformations, fatigue, or microscale failures do not occur over longer periods of operation.

Future work should focus on addressing these shortcomings, as well as expanding the scope of the research to include multiple other forms of FMIs. Pistons, as suggested by Giunta et al. [23], should also be investigated as potential FMIs due to their improved resistance to deformation at higher pressures. However, this comes with increased mass, potentially making them less efficient at higher frequencies or with smaller PtP pressures. Additionally, this research only focused on the use of steel diaphragms; future research should attempt to investigate the performance of other materials, such as composites.

## 5. Conclusions

This paper investigates the relationship between the thickness of a diaphragm used as a fluid-mechanical interface (FMI) to determine how varying said thickness impacts the transmission of force from the hydraulic fluid into the piezoelectric stack. The study presented in this paper has highlighted that a diaphragm thickness of up to 0.8 mm is viable for sensing applications where the transmissivity of force is less relevant. Additionally, the study highlighted that a 0.25 mm diaphragm appears to be the best thickness for energy harvesting applications as it guarantees safety while still transmitting force essentially as well as the notably thinner 0.1 mm diaphragm as highlighted in Figure 7. This is true for a 48 mm diameter diaphragm in stainless steel as this was the type of diaphragm used in the experiments presented in this study. Furthermore, the peak pressure used in this study was 50 bar; thicker diaphragms will be required for higher-pressure applications. Moreover, cyclical testing would need to be undertaken to guarantee the long-term integrity of the diaphragm.

## Data Availability

All data contained within article.
